# Supplementary material for: An open-source framework for large-scale, flexible evaluation of biomedical text mining systems
Source: J Biomed Discov Collab. 2008 Jan 29;3:1. doi: 10.1186/1747-5333-3-1 (PMC2276192; doi:10.1186/1747-5333-3-1)
Supplement: Additional file 1 — Evaluation platform source code. The additional file contains the software evaluation framework discussed in this paper. The most current release can be downloaded from SourceForge.net [1]. See the accompanying README file for instructions on installation and use. Also included in the distribution is a collection of UIMA wrappers for some commonly used BioNLP tools and annotated corpora. [file 1747-5333-3-1-S1.zip › bionlp_uima_repository-1.0.1/docs/api/edu/uchsc/ccp/uima/util/opendmap/TestSummarizer.html]

TestSummarizer (Center for Computational Pharmacology Util/UIMA API)


|  |  |  |
| --- | --- | --- |
| **Overview** \| **Package** \| **Class** \| **Tree** \| **Deprecated** \| **Index** \| **Help** | |  |
| **PREV CLASS**   NEXT CLASS | **FRAMES**    **NO FRAMES**     **All Classes** |  |
| SUMMARY: NESTED \| FIELD \| CONSTR \| METHOD | DETAIL: FIELD \| CONSTR \| METHOD |  |


---


## edu.uchsc.ccp.uima.util.opendmap Class TestSummarizer

```
java.lang.Object
  edu.uchsc.ccp.uima.util.opendmap.TestSummarizer
```

---

``` public class TestSummarizer extends Object ```

This class prints out selected contents of the CAS in the standard
annotation summery format.

**Author:**
:   R. James Firby

---

**Constructor Summary**

`TestSummarizer(String mentionPattern)`
  
          Create a new TestSummarizer that will generate summary strings for the mentions and slots
specified in the pattern.


| **Method Summary** | |
| --- | --- |
| `ArrayList<String>` | `summarizeJCas(String docId, org.apache.uima.jcas.JCas jcas)`             Generate summary strings for all annotations in the JCAS that are described in the pattern string for this summarizer. |

**Methods inherited from class java.lang.Object**

`clone, equals, finalize, getClass, hashCode, notify, notifyAll, toString, wait, wait, wait`

**Constructor Detail**

### TestSummarizer

```
public TestSummarizer(String mentionPattern)
```

:   Create a new TestSummarizer that will generate summary strings for the mentions and slots
    specified in the pattern.

    A mention pattern has the form:

    ```
       type,slot,slot;type,slot,slot;...
    ```

    Where 'type' is the name of a mention type to print, and the 'slot's
    following the 'type' are thje slots for that type to include in the
    output.

    **Parameters:**: `mentionPattern` - The pattern defining the mention types and slots to print.


**Method Detail**

### summarizeJCas

```
public ArrayList<String> summarizeJCas(String docId,
                                       org.apache.uima.jcas.JCas jcas)
```

:   Generate summary strings for all annotations in the JCAS that are described in the
    pattern string for this summarizer.

    :   **Parameters:**: `docId` - The document ID that has been analyzed to fill the JCas: `jcas` - The JCas holding the annotations to be summarized **Returns:**: A set of summary strings, one for each annotation of interest in the JCas


---


|  |  |  |
| --- | --- | --- |
| **Overview** \| **Package** \| **Class** \| **Tree** \| **Deprecated** \| **Index** \| **Help** | |  |
| **PREV CLASS**   NEXT CLASS | **FRAMES**    **NO FRAMES**     **All Classes** |  |
| SUMMARY: NESTED \| FIELD \| CONSTR \| METHOD | DETAIL: FIELD \| CONSTR \| METHOD |  |


---
